# The potential role of BCL-2 inhibition in amyloidosis and plasma cell leukemia

**DOI:** 10.3389/fonc.2025.1549891

**Published:** 2025-03-21

**Authors:** Grigorios Alvanidis, Dimitrios Kotsos, Christina Frouzaki, Amalia Fola, Evdoxia Hatjiharissi

**Affiliations:** Division of Hematology, 1st Department of Internal Medicine, Aristotle University of Thessaloniki, AHEPA University Hospital, Thessaloniki, Greece

**Keywords:** BCL-2 inhibition, venetoclax, plasma cell neoplasms, plasma cell leukemia, AL amyloidosis, hematologic malignancies

## Abstract

Plasma cell neoplasms include a spectrum of disorders, such as plasma cell leukemia (PCL) and light chain (AL) amyloidosis, all associated with poor prognosis and limited therapeutic options. Venetoclax is the first-in-class B-cell lymphoma 2 (BCL-2) inhibitor and triggers apoptosis selectively in cells reliant on the BCL-2 pathway for survival. Randomized clinical trials have established the anti-tumor activity and efficacy of venetoclax in selected patients with hematologic malignancies such as acute myeloid leukemia (AML), chronic lymphocytic leukemia (CLL), and multiple myeloma (MM). At the same time, recent studies suggest its potential application in rare plasma cell dyscrasias. Preliminary results from case reports and a small cohort of patients indicate that venetoclax may benefit patients with PCL. Regimens incorporating venetoclax have also demonstrated promising outcomes in patients with AL amyloidosis, particularly those with translocation (11;14). This review analyzes new data on venetoclax in AL amyloidosis and PCL and highlights the increasing significance of BCL-2 inhibition in plasma cell neoplasms beyond MM.

## Introduction

B-cell lymphoma 2 (BCL-2) is an essential apoptosis regulator instrumental in cell survival by inhibiting programmed cell death. The expression of BCL-2 is often dysregulated in hematologic malignancies where cells frequently evade apoptosis, rendering it a possible target for therapy. Venetoclax, the first-in-class drug of BCL-2 inhibitors, selectively triggers apoptosis in cells dependent on BCL-2 for survival and has demonstrated favorable outcomes in patients with a diverse spectrum of hematologic neoplasms, including chronic lymphocytic leukemia (CLL), acute myeloid leukemia (AML), and multiple myeloma (MM) (1–5). As a result, venetoclax represents a promising targeted agent for other rare and challenging plasma cell neoplasms, including light chain (AL) amyloidosis and plasma cell leukemia (PCL) (6, 7).

AL amyloidosis and PCL constitute a heterogeneous biological and clinical spectrum of plasma cell conditions, typically associated with a poor prognosis and characterized by a lack of effective therapy options. A small in size but dangerous clonal population characterizes AL amyloidosis, whereas PCL is distinguished by an excessive proliferation of plasma cells that grow independently from the bone marrow microenvironment (8–10). Despite the development of numerous innovative targeted and immunotherapeutic treatments, which have greatly improved overall survival (OS) for patients with MM, both of these rare diseases continue to exhibit unfavorable outcomes, particularly for individuals ineligible for stem cell transplantation.

Interest in BCL-2 inhibition has increased, especially for patients with the t(11;14) translocation, a cytogenetic marker found in over 50% of individuals with AL amyloidosis and PCL, which is linked to and promotes BCL-2 activation (11, 12). Preliminary data from several case reports and small cohort studies suggest that venetoclax may substantially impact responses in these patients. Nevertheless, these findings require additional clinical validation in larger cohorts of individuals.

This review will explore the evolving role of BCL-2 inhibition in AL amyloidosis and PCL, with a particular focus on venetoclax, by synthesizing existing data and identifying knowledge gaps, thereby offering insights to guide future research and clinical practice in these difficult-to-treat malignancies.

## AL amyloidosis

AL amyloidosis is the most common and severe form of amyloidosis. It is caused by the synthesis and deposit of abnormal, misfolded kappa or lambda light chains in the tissues (13–15), which over time leads to amyloid accumulation causing organ damage and dysfunction. The heart, kidneys, liver, and nervous system are major sites of clinically significant deposition. Prognosis is poor, as indicated by 1-year and 5-year relative survival rates of 79% and 43%, respectively (16). The severity of organ involvement impacts the prognosis, with the primary determinant of outcome remaining the extent of cardiac involvement (17).

Over the past years, the use of novel agents, timely diagnosis, and refined selection criteria for patients undergoing autologous stem cell transplant (ASCT) have all contributed to improving outcomes for patients with AL amyloidosis. At the same time, the role of ASCT is being challenged by the emergence of new therapeutic strategies. The treatment of AL amyloidosis developed from protocols originally adapted for MM, as the primary objective of treatment is to reduce the plasma cell clone (18, 19). In 2021, the US Food and Drug Administration approved daratumumab in combination with cyclophosphamide, bortezomib, and dexamethasone as the initial treatment for AL amyloidosis. This combination has since become the standard of care for patients who are not eligible for transplantation (18). However, the treatment of AL amyloidosis is generally guided by the patient’s risk profile. The extent of organ involvement, performance status, and age determine the selection of therapeutic agents and treatment intensity at diagnosis and in the relapsed setting.

Venetoclax has emerged as a promising therapeutic candidate due to its efficacy in patients with MM who have t(11;14), as approximately 50% of patients with AL amyloidosis also have this translocation (12, 20). Due to the lack of outcomes from prospective trials, most investigations assessing the efficacy and safety of venetoclax-based regimens for AL amyloidosis have relied on retrospective analyses in the relapsed or refractory context of the disease.

### The efficacy and toxicity of venetoclax in AL amyloidosis

Dima et al. recently published a study demonstrating the efficacy of venetoclax in patients with t(11;14) AL amyloidosis who were refractory to daratumumab-based regimens (6). This retrospective analysis included 31 patients and showed the significant efficacy of venetoclax-based therapy, achieving an overall hematologic response rate of 97% and a very good partial response (VGPR) in over 91% of the patients. Additionally, 14 of the 19 evaluable patients with cardiac involvement exhibited an organ response, while 6 of the 13 patients with renal involvement also demonstrated an organ response. The study indicated a favorable safety profile for venetoclax, with severe (grade ≥3) adverse events occurring in 26% of the patients and infections accounting for 6%. The OS rates at 12 and 24 months were 89% and 85%, respectively (6).

Another multicenter retrospective study investigated the efficacy of venetoclax in 26 relapsed/refractory (R/R) AL patients previously treated with daratumumab, with 88% having the t(11;14) aberration (21). The study showed an overall hematologic response rate of 88%, with 35% (9/26) of patients attaining complete remission (CR), another 35% (9/26) achieving VGPR, and 19% (5/26) reaching partial response (PR). The study reported a median time to best response of 2 months (range 0.3–11 months) and the median OS was 33 months (95% confidence interval (CI), 25.9-39.2 months). The treatment regimen had a rather safe profile, with 3 (11%) documented grade 3-5 infections and 4 (15%) grade 3-4 cytopenias (21).

Recently, Roussel et al. reported the findings of a retrospective study from the French Amyloidosis Network that included 51 patients who received venetoclax (22). Patients received venetoclax as a single agent or as part of therapeutic regimens that included dexamethasone, bortezomib, and daratumumab. Twenty-five patients were treated solely with venetoclax (100-800 mg/daily), 5 patients received venetoclax with dexamethasone, 12 patients were given venetoclax with bortezomib, 3 patients were given venetoclax and daratumumab and 6 patients received all four drugs. The hematologic response rate for this study was 90%, as 61% achieved CR, 14% reached VGPR and 10% had PR. In contrast, only one of the three patients lacking t(11;14) reached CR. The administration of venetoclax demonstrated favorable tolerability, with 18 patients experiencing neutropenia, diarrhea, nausea, infections, and fatigue (22). Two other retrospective studies have reported comparable efficacy outcomes in AL amyloidosis patients resistant to previous therapies and harboring t(11;14), aligning with the results of the French Amyloidosis Network research (23, 24). Both studies demonstrated that venetoclax-based regimens can elicit hematologic and organ responses, particularly in the heart and kidneys, while exhibiting acceptable toxicity.

Premkumar et al. enrolled 43 patients with previously treated AL from 14 centers across the US and Europe. The study showed the efficacy of venetoclax combined with glucocorticoids and/or proteasome inhibitors, with venetoclax dosages ranging from 100 to 800 mg daily (23). The median follow-up time was 14.5 months, whereas the median time to best response was 8.5 weeks. The best response (VGPR/CR) in patients with t(11;14) (27 patients) was higher when compared to the best response of all evaluable patients (38 patients), with rates of 78% and 63%, respectively. Interestingly, patients with t(11;14) had a significant 86% reduction in the probability of progression or death. The safety assessment indicated that 8 of the 43 patients (19%) experienced grade ≥3 therapy-related non-hematologic side effects, and 8 of 43 patients (19%) discontinued treatment due to therapy-related toxicity (23).

Sidiqi et al. administered venetoclax either as a single agent (5/12 patients) or combined with dexamethasone (2/12 patients), with bortezomib and dexamethasone (3/12), with bortezomib, cyclophosphamide, and dexamethasone (1/12), and with bortezomib, lenalidomide, and dexamethasone (1/12) (24). The treatment regimen for venetoclax differed, with 7 patients receiving 800 mg daily and 5 patients receiving 400 mg daily, while 8 patients commenced with lower doses that were progressively escalated to the final target dose. The median follow-up time was 11.5 months (95% CI, 2-21 months), and the median duration of therapy was 5 months (range 1-27 months). The median time to best hematologic response was 3.4 months (range 1.6-8.4 months). Among the 8 patients evaluated for hematologic response, 4 attained CR, 3 obtained VGPR, and 1 had no response to treatment. Six patients exhibited moderate gastrointestinal problems, and three patients terminated therapy: two due to side effects (cytopenia and dyspnea) and one due to insufficient response to treatment (24).

Rieger et al. reported the findings of a three-year follow-up including 9 R/R AL amyloidosis patients with t(11;14) (25). Venetoclax-based treatment induced rapid hematologic responses, with the initial response recorded at 26 days (range 11-125 days) and the best response after 106 days (range 35-659). The study demonstrated significant efficacy, reporting an overall response rate (ORR) of 100%. Specifically, 7 out of 9 patients achieved CR, and 2 out of 9 achieved VGPR. Cardiac responses occurred in 6 out of 9 patients, while renal responses were observed in 3 out of 5. A study of 21 patients with t(11;14) AL amyloidosis, refractory to daratumumab, indicated that venetoclax administered in various combinations could achieve rapid responses (26). The ORR was 95%, with a median time to hematologic response of 2 months (range 1–5 months). Ventetoclax was administered in combination primarily with dexamethasone (9/21 patients), dexamethasone and daratumumab (6/21 patients), or dexamethasone and a proteasome inhibitor (6/21 patients). The progression-free survival (PFS) rates at 6 months and 12 months were 83% and 68%, respectively, while the OS rates at the same intervals were 89% and 83%, respectively (26).

### Recruiting/ongoing clinical trials and future directions

Current investigations into venetoclax-based treatment protocols for AL amyloidosis target patients with or without t(11;14). These trials aim to determine the safety, effectiveness, maximum tolerated dose (MTD), and recommended phase 2 dose (RP2D) of venetoclax in combination with various therapeutics. Lu et al. are conducting a trial (NCT06192979) that aims to identify the optimal first-line treatment for AL amyloidosis patients with t(11;14), using an initial regimen containing daratumumab, bortezomib, and dexamethasone (DBD). Patients who do not attain a hematologic response within 7 days post-DBD will be administered venetoclax in conjunction with daratumumab and dexamethasone. Ongoing recruiting trials are investigating venetoclax in combination with dexamethasone (NCT05451771), with ixazomib and dexamethasone (NCT04847453), and with daratumumab and dexamethasone (NCT05486481) (**Table 1**).

**Table 1:** Active and recruiting clinical trials for AL amyloidosis (according to https://clinicaltrials.gov/).

Trial number	Condition	Regimen	Phase	Location
NCT06192979	AL Amyloidosis with t(11; 14)	Venetoclax + Daratumumab + Bortezomib + Dexamethasone	–	Peking University People’s Hospital, Beijing, China
NCT05451771	R/R AL Amyloidosis with t(11;14)	Venetoclax + Dexamethasone	I/II	Multicenter Study, United States
NCT04847453	R/R AL Amyloidosis	Venetoclax + Dexamethasone + MLN9708 (ixazomib citrate)	I/Ia	Multicenter Study, United States
NCT05486481	AL Amyloidosis with t(11;14)	Venetoclax + Daratumumab + Dexamethasone	I/II	University of California, San Francisco, United States

AL amyloidosis, light chain amyloidosis; R/R, relapsed/refractory; t(11;14), translocation 11;14.

Lastly, Kastritis et al. aims to assess the safety and effectiveness of ZN-d5, a novel and selective BCL-2 inhibitor, in R/R AL amyloidosis patients (27). Phase I of the study will focus on identifying the safety profile, tolerability, MTD, and RP2D of ZN-d5. Patients are expected to receive escalating doses of the drug (from 200 up to 1600 mg daily), while Phase II aims to evaluate the hematological response rate in patients with or without t(11; 14).

Venetoclax-based therapies have induced rapid hematologic and organ responses in patients with AL amyloidosis, particularly those with t(11;14). Retrospective studies have shown that venetoclax is an effective treatment for patients who failed prior therapies and provide preliminary evidence of its safety and effectiveness in AL amyloidosis.

## Plasma cell leukemia

PCL is the most aggressive form of plasma cell neoplasms (9, 28), characterized by the presence of ≥ 5% of circulating plasma cells in the peripheral blood smear based on the new, revised definition (29). It is subdivided into primary (pPCL) when the leukemic phase is evident at the time of diagnosis and secondary PCL (sPCL) when the leukemic transformation occurs in the setting of a pre-existing R/R MM (9, 28, 30). Therapeutic options for PCL, particularly pPCL, remain limited, with intensive conventional chemotherapy being the most commonly used first-line treatment. However, the prognosis in PCL is dismal, with patients having a median OS of only 7 months after receiving conventional chemotherapy (30). Identifying novel and effective therapeutic modalities remains an unmet need in the treatment of PCL.

### The efficacy and toxicity of venetoclax in PCL

Venetoclax has demonstrated significant effectiveness in managing R/R multiple myeloma. The efficacy is closely linked to the existence of t(11;14), prompting attention to its potential applicability for patients with PCL, who display the t(11;14) in 25-65% of cases (9, 11, 31). Currently, scant research has evaluated the efficacy and safety of venetoclax in patients with PCL, mostly through case reports.

The first report of the use of BCL-2 inhibitors in refractory pPCL was published in 2017, describing a case of a patient with t(11;14). The patient underwent three treatment regimens, including carfilzomib, lenalidomide, and dexamethasone, followed by VDT-PACE and ASCT, along with daratumumab, doxorubicin, cyclophosphamide, and dexamethasone due to transient responses. After these treatments, the disease recurred, prompting the initiation of a weekly regimen comprising daratumumab, bortezomib, venetoclax (800 mg daily), and dexamethasone. This regimen achieved a significant hematologic response after three months, and at the time of publication, the patient remained in remission while receiving this quadruplet therapy (32).

Subsequently, seven case reports have demonstrated encouraging outcomes from regimens employing venetoclax for the treatment of pPCL patients with t(11;14) (33–38) and those without (39), resulting in deep responses with CR and VGPR (6/7 cases), while only one case did not achieve a response (38). The venetoclax dose in the reported case reports varied, with the minimum dosage recorded at 100 mg daily (35, 38) and the maximum at 600 mg (36). The most commonly prescribed dosages were 300 mg (33, 39) and 400 mg (34, 37). sPCL also has a poor prognosis, mostly because it emerges in end-stage MM patients who have undergone extensive prior treatment (30). The effectiveness of venetoclax-based therapy in inducing hematologic responses, including VGPR and CR, has been evidenced in three case studies involving R/R MM patients diagnosed with sPCL and t(11;14) (40–42).

Szita et al. reported the largest cohort of refractory PCL patients effectively treated with venetoclax in a single study (7). The study presented the outcomes of 58 MM patients with t(11;14) treated with venetoclax in seven Hungarian centers, including 4 sPCL patients and 2 pPCL patients. Patients in the R/R or reinduction settings, following an unsatisfactory response to frontline treatment, received venetoclax. The results indicated significant efficacy of venetoclax therapy in patients with PCL, with all patients achieving a response greater than PR. Patients in the R/R setting achieved notable outcomes, with a PFS of 10 months and an OS of 12.2 months (7). Concerning adverse events recorded in PCL patients, 67% experienced infections after venetoclax therapy, resulting in one death, while 83% of treated patients developed cytopenias of varying severity (7).

The therapeutic landscape of PCL, especially for patients with t(11;14), is constantly evolving, with published studies demonstrating the promising potential of BCL-2 inhibitors like venetoclax. A more comprehensive knowledge of the efficacy of venetoclax is required, as there have been reports of its inability to prevent disease progression in PCL patients, both with and without the presence of t(11;14) (43, 44). Most data in the literature regarding both pPCL and sPCL consist predominantly of case reports, a reflection of the disease’s aggressive and rare characteristics. Given that a viable treatment for PCL is still lacking, additional clinical trials are necessary to validate the previously described findings and investigate the efficacy and safety of BCL-2 inhibition in the context of PCL.

## Discussion

BCL-2 inhibition, primarily with venetoclax, represents a promising therapeutic option for plasma cell neoplasms beyond MM. Venetoclax induces hematologic and organ responses in AL amyloidosis and shows benefits in extensively treated patients with PCL. Despite the limited data, numerous case reports and retrospective studies demonstrated significant efficacy, particularly evident in the cohort of individuals with t(11;14), a biomarker distinctly associated with better response to venetoclax in MM, as shown by the results of the BELLINI study (45). The benefit of venetoclax treatment for patients with non-t(11;14) AL, who undoubtedly have less data compared to t(11;14) individuals, needs to be further evaluated. However, several other important questions also remain unanswered regarding the role of BCL-2 inhibition in rare plasma cell dyscrasias, such as AL amyloidosis and PCL.

To optimize therapeutic benefit and minimize toxicity, disease-specific dosage of venetoclax must be defined. The ideal dosage for AL amyloidosis and PCL is unclear, although daily dosages of venetoclax up to 400 mg instead of 800 mg in the setting of MM seems to be effective (46). In AL amyloidosis, managing organ involvement and toxicity is critical; thus, dose adjustments that favor safety may be required. In contrast, the aggressive nature of PCL may necessitate higher doses due to its rapid progression.

The optimal drug combinations for venetoclax remain also undetermined and may vary considerably between AL amyloidosis and PCL because of their distinct biological characteristics. Venetoclax has potential in patients with R/R AL amyloidosis following daratumumab failure (6). Additionally, for patients with t(11;14), it may be incorporated into the daratumumab, cyclophosphamide, bortezomib, and dexamethasone regimen, which is the standard of care treatment for patients with AL amyloidosis (18). Preliminary evidence indicates that venetoclax, in conjunction with proteasome inhibitors or immunomodulatory agents, may produce favorable outcomes for PCL. Nonetheless, the scarcity of PCL necessitates multidisciplinary cooperation to recruit adequate patient populations for comprehensive research.

Although studies have demonstrated promising results, they have also reported grade ≥3 adverse events including infections, neutropenia, and gastrointestinal symptoms. Clinicians should be prepared to manage cytopenias, which are common side effects, especially in heavily pretreated patients (47). Moreover, venetoclax carries the risk of tumor lysis syndrome (48–50), particularly in patients with a high tumor burden, such as those with PCL. Close monitoring, antibiotics, oral and intravenous hydration, and gradual increase of the drug dosage over time are essential to prevent and manage these adverse events.

In conclusion, prospective trials are needed to corroborate the favorable outcomes observed, since the information on the efficacy of BCL-2 inhibitors in PCL and AL amyloidosis is based on small cohorts or case reports. The heterogeneity of those rare conditions indicates that BCL-2 inhibition will not be effective for every case. Nevertheless, it is essential to investigate improved combination regimens and predictive biomarkers, particularly focusing on BCL-2 expression, in future trials. Moreover, additional research is required to determine the appropriate and safe dose of venetoclax in this patient population.
